# Distribution of transpulmonary pressure during one-lung ventilation in pigs at different body positions

**DOI:** 10.3389/fphys.2023.1204531

**Published:** 2023-08-04

**Authors:** Jakob Wittenstein, Martin Scharffenberg, Xiuli Yang, Thomas Bluth, Thomas Kiss, Marcus J. Schultz, Patricia R. M. Rocco, Paolo Pelosi, Marcelo Gama de Abreu, Robert Huhle

**Affiliations:** ^1^ Department of Anesthesiology and Intensive Care Medicine, Pulmonary Engineering Group, University Hospital Carl Gustav Carus Dresden at Technische Universität Dresden, Dresden, Germany; ^2^ Department of Anesthesiology, First Affiliated Hospital of Anhui Medical University, Hefei, China; ^3^ Department of Anaesthesiology, Intensive-Pain- and Palliative Care Medicine, Radebeul Hospital, Academic Hospital of the Technische Universität Dresden, Radebeul, Germany; ^4^ Department of Intensive Care and Laboratory of Experimental Intensive Care and Anaesthesiology, Academic Medical Center, University of Amsterdam, Amsterdam, Netherlands; ^5^ Laboratory of Pulmonary Investigation, Carlos Chagas Filho Institute of Biophysics, Federal University of Rio de Janeiro, Rio de Janeiro, Brazil; ^6^ Department of Surgical Sciences and Integrated Diagnostics, University of Genoa, Genoa, Italy; ^7^ Anesthesia and Critical Care, San Martino Policlinico Hospital, IRCCS for Oncology and Neurosciences, Genoa, Italy; ^8^ Department of Intensive Care and Resuscitation, Anesthesiology Institute, Cleveland Clinic, Cleveland, OH, United States; ^9^ Department of Outcomes Research, Anesthesiology Institute, Cleveland Clinic, Cleveland, OH, United States

**Keywords:** OLV, VILI, thoracic surgery, local transpulmonary pressure, local pleural pressure, mechanical power, open pneumothorax

## Abstract

**Background**. Global and regional transpulmonary pressure (P_L_) during one-lung ventilation (OLV) is poorly characterized. We hypothesized that global and regional P_L_ and driving P_L_ (ΔP_L_) increase during protective low tidal volume OLV compared to two-lung ventilation (TLV), and vary with body position.

**Methods**. In sixteen anesthetized juvenile pigs, intra-pleural pressure sensors were placed in ventral, dorsal, and caudal zones of the left hemithorax by video-assisted thoracoscopy. A right thoracotomy was performed and lipopolysaccharide administered intravenously to mimic the inflammatory response due to thoracic surgery. Animals were ventilated in a volume-controlled mode with a tidal volume (V_T_) of 6 mL kg^−1^ during TLV and of 5 mL kg^−1^ during OLV and a positive end-expiratory pressure (PEEP) of 5 cmH_2_O. Global and local transpulmonary pressures were calculated. Lung instability was defined as end-expiratory P_L_<2.9 cmH_2_O according to previous investigations. Variables were acquired during TLV (TLVsupine), left lung ventilation in supine (OLVsupine), semilateral (OLVsemilateral), lateral (OLVlateral) and prone (OLVprone) positions randomized according to Latin-square sequence. Effects of position were tested using repeated measures ANOVA.

**Results**. End-expiratory P_L_ and ΔP_L_ were higher during OLVsupine than TLVsupine. During OLV, regional end-inspiratory P_L_ and ΔP_L_ did not differ significantly among body positions. Yet, end-expiratory P_L_ was lower in semilateral (ventral: 4.8 ± 2.9 cmH_2_O; caudal: 3.1 ± 2.6 cmH_2_O) and lateral (ventral: 1.9 ± 3.3 cmH_2_O; caudal: 2.7 ± 1.7 cmH_2_O) compared to supine (ventral: 4.8 ± 2.9 cmH_2_O; caudal: 3.1 ± 2.6 cmH_2_O) and prone position (ventral: 1.7 ± 2.5 cmH_2_O; caudal: 3.3 ± 1.6 cmH_2_O), mainly in ventral (*p* ≤ 0.001) and caudal (*p* = 0.007) regions. Lung instability was detected more often in semilateral (26 out of 48 measurements; *p* = 0.012) and lateral (29 out of 48 measurements, *p* < 0.001) as compared to supine position (15 out of 48 measurements), and more often in lateral as compared to prone position (19 out of 48 measurements, *p* = 0.027).

**Conclusion**. Compared to TLV, OLV increased lung stress. Body position did not affect stress of the ventilated lung during OLV, but lung stability was lowest in semilateral and lateral decubitus position.

## 1 Introduction

One-lung ventilation (OLV) for thoracic surgery leads to pronounced changes in respiratory system mechanics. When the thorax is opened and lung isolation achieved, not only absolute values, but also the distribution of intrathoracic pressure may change importantly. Furthermore, lateral decubitus position, which is commonly used during thoracic surgery, can promote atelectasis formation, and reduce the total lung volume. As a result, both global and regional mechanical stress on lung parenchyma may increase and lead to ventilator-induced lung injury (VILI) (Slutsky and Ranieri, 2013). In fact, the risk of VILI is considered to be higher during OLV than two-lung ventilation (TLV) (Lohser and Slinger, 2015).

Currently, respiratory system mechanics and mechanical power (MP), as well as global regional end-inspiratory and end-expiratory transpulmonary pressure (P_L_), and driving P_L_ (ΔP_L_) during OLV are poorly defined. Theoretically, higher end-inspiratory P_L_ might increase stress and overdistend the lungs, whereas negative end-expiratory P_L_ might cause instability with tidal collapse and reopening of lung units. Also, excessive transpulmonary driving pressure (ΔP_L_) could further increase lung stress and injury (Grieco et al., 2017). To prevent this, V_T_ during OLV should not exceed 6 mL kg^−1^ predicted body weight (Batchelor et al., 2019).

In this study, we aimed at determining P_L_ and ΔP_L_ in different lung regions, as well as the elastance, resistance and MP of the respiratory system during OLV in anesthetized pigs. We hypothesized that, compared to TLV, OLV increases P_L_ and ΔP_L_ and impairs respiratory system mechanics and MP. Also, we hypothesized that the gravity gradient is higher in OLV and, thus, OLV in lateral position increases stress and instability of the ventilated lung compared to semi-lateral, supine and prone positions.

## 2 Methods

This study was conducted as a secondary protocol of a previous experiment from our group (Wittenstein et al., 2021), and was approved by the Institutional Animal Care and Welfare Committee and the Government of the State of Saxony, Germany (DD24.1-5131/449/71). In the primary study, we determined the distribution of pulmonary blood flow during commonly used body positions for thoracic surgery during intravascular normo- and hypovolemia (n per group = 8) in pigs undergoing OLV (Wittenstein et al., 2021). All animals received care in compliance with the Principles of Laboratory Animal Care formulated by the National Society for Medical Research and the US National Academy of Sciences Guide for the Care and Use of Laboratory Animals, and complied with relevant aspects of the ARRIVE guidelines. Animals were kept at controlled temperature and light-dark cycle with free access to water and food.

### 2.1 Experimental protocol

The animal preparations were described in detail elsewhere (Wittenstein et al., 2021). Briefly, intravenous anesthesia and muscle paralysis were induced and maintained with midazolam (1 mg kg^-1^ h^-1^), ketamine (15 mg kg^-1^ h^-1^), and atracurium (3 mg kg^− 1^ h^-1^) in sixteen female pigs (German landrace, weighing 35–49 kg, Danish Specific Pathogen Free Certification (Health Status Management, 2022)). The intravascular volume was maintained with a crystalloid solution at a rate of 5 mL kg^-1^ h^-1^. Norepinephrine was used to maintain a mean arterial pressure of at least 60 mmHg throughout the experiments. Lungs were ventilated in volume-controlled mode: fraction of inspired oxygen (F_I_O_2_) of 1.0, tidal volume (V_T_) of 6 mL kg^-1^, positive end-expiratory pressure (PEEP) of 5 cmH_2_O, inspiratory: expiratory (I:E) ratio of 1:1, the constant gas flow of 25 L min^-1^, and respiratory rate (RR) adjusted to achieve arterial pH > 7.3 (Evita XL, Drägerwerk AG & Co. KGaA, Lübeck, Germany).

All skin incisions were preceded by infiltration with 2–5 mL lidocaine 2%. A 20 cm PiCCO catheter was inserted in the right internal carotid artery. A 7.5 Fr. pulmonary artery catheter was advanced through an 8.5 Fr. sheath, placed in the right external jugular vein until typical pulmonary arterial pressure waveforms were observed. Urine was collected with a bladder catheter inserted through a median mini-laparotomy.

Regional pleural pressures (P_Pl_) were measured using three custom-made pressure sensors, calibrated and placed as previously described by our group (Kiss et al., 2019). Briefly, pressure sensors were made from a glued (Silicon-based clue, MBFZ toolcraft GmbH, Georgensgmünd, Germany) double layer of thin latex to build a sealed air-filled chamber (30 × 30 × 3 mm^3^). A silicon tube (50 cm length, 500 µm inner diameter, 1.2 mm outer diameter) was introduced into the chamber using seldinger technique and connected to the respective pressure transducers (163PC01D48-PCB, FirstSensors AG, Berlin, Germany) via Luer-lock. Before implantation, each sensor was inflated with 0.05 mL of room air and calibrated within an air-sealed chamber using pressures in the range of −50 to 50 cmH_2_O within 30 s, yielding a pressure rate of 6.7 cmH_2_O s^-1^. For calibration, a linear correction was used.

**FIGURE 1:**
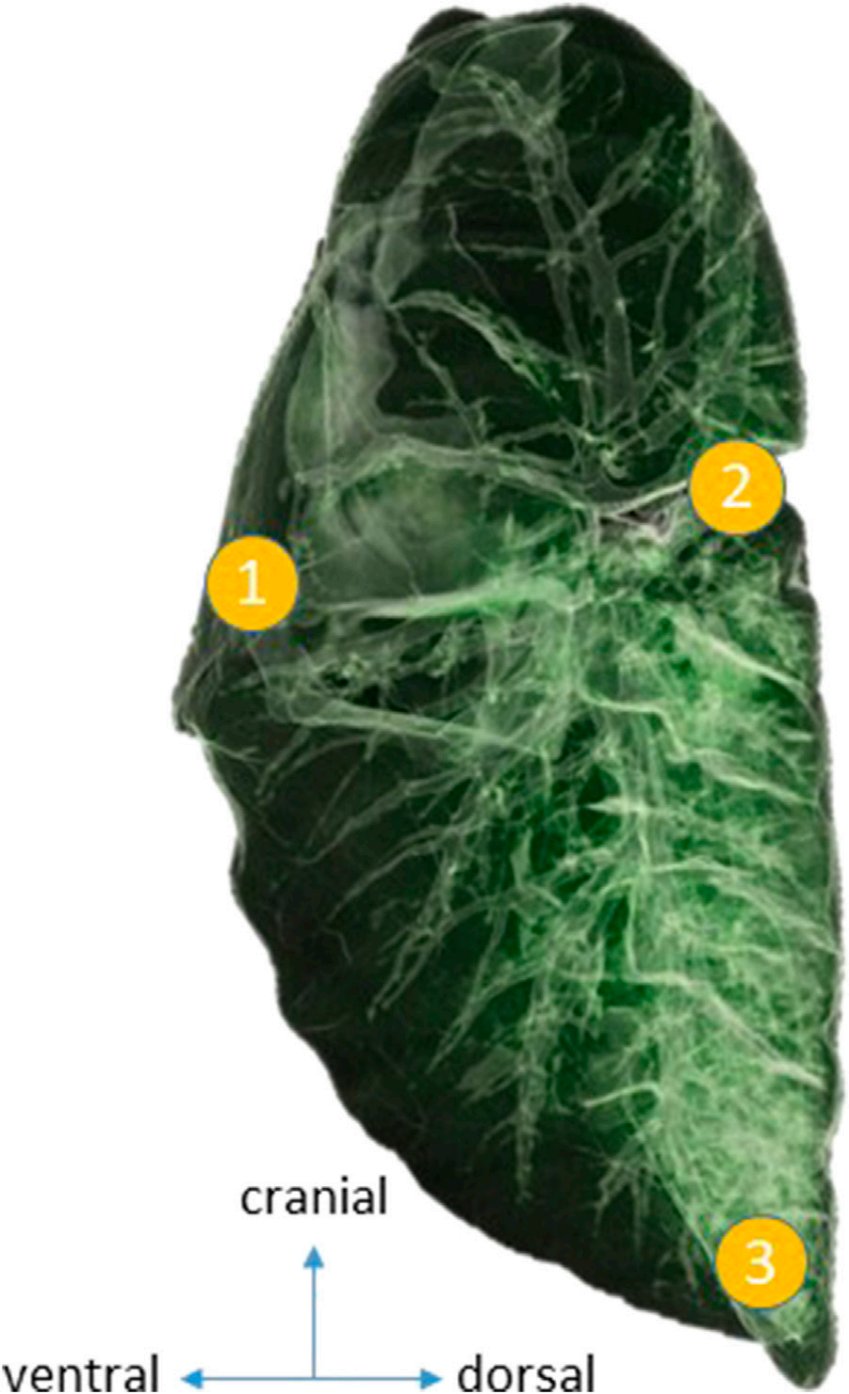
Position of the intrapleural pressure sensors. To measure regional pleural pressure, three pressure sensors were custom-made, calibrated, and placed via video-assisted thoracoscopy. The sensors were attached to the parietal pleura by staples in the following regions of the left hemithorax: 1) ventral (4–5th rib parasternal); 2) dorsal (4–5th rib paravertebral); and 3) caudal (8–9th rib paravertebral).

For placement of the sensors, lungs were separated introducing a left-sided double-lumen tube (39 Fr., Silbroncho Fuji, Tokyo, Japan) through a tracheotomy, with the bronchial tip placed into the left main bronchus under fiberoptic control (Ambu aScope 3 and Ambu aView, Ambu GmbH, Bad Nauheim, Germany). Animals were positioned in the right lateral decubitus position, and OLV of the dependent lung (V_T_ = 5 mL kg^-1^, RR = 35 min^-1^) was initiated. Using video-assisted thoracoscopy (Thoracoscopy Set, Karl Storz, Tuttlingen, Germany), three pressure sensors were attached to the parietal pleura by staples in the following regions of the left hemithorax: 1) ventral (4–5th rib parasternal); 2) dorsal (4–5th rib paravertebral); and 3) caudal (8–9th rib paravertebral) (Figure 1). A thoracic drain was placed, and surgical wounds were sutured and sealed.

To mimic thoracic surgery conditions, a right-sided thoracotomy was performed between the medial-clavicular and the anterior axillary line in the fourth-fifth intercostal space, and a rib spreader was placed. Furthermore, systemic inflammation was induced by continuous administration of 0.5 μg kg^-1^ h^-1^ Lipopolysaccharides (LPS) from *Escherichia coli* O111:B4 (SIGMA Aldrich, St. Louis, United States) through the central venous line.

According to the protocol of the primary study, animals were randomized to either normo- or the hypovolemia group. For induction of hypovolemia, 25% of the calculated blood volume, estimated as 70 mL kg^-1^. However, in this substudy all animals were analysed independently from their volume status. Animals were randomly submitted to one of four sequences during OLV according to a Latin square cross-over design: 1) a-b-c-d, 2) b-d-a-c, 3) d-c-b-a, and 4) c-a-d-b (with a = supine; b = left semilateral; c = left lateral; d = prone position; 30 min each). For protective low tidal volume OLV, volume-controlled mode was used (V_T_ = 5 mL kg^-1^; F_I_O_2_ = 1.0, PEEP = 5 cmH_2_O, I:E = 1:1, RR = 30–35 min^-1^ to achieve arterial pH > 7.25, and gas flow = 25 L min^-1^). To reset lung history between the interventions, animals were placed in the supine position and disconnected from the ventilator (20 s), TLV resumed with baseline settings until normalization of gas exchange, and an alveolar recruitment maneuver performed before the start of OLV in each position.

### 2.2 Measurements

P_L_ and respiratory variables were first collected after completing instrumentation (Baseline). Measurements were then repeated 1 h after starting the LPS infusion (TLVsupine) and 30 min after turning the animal into each body position during OLV (OLVsupine, OLVsemilateral, OLVlateral, and OLVprone, 30 min each).

#### 2.2.1 Respiratory signals, pleural pressures and regional P_L_

Signals were recorded for 5 minutes at each time point. Air flow and airway pressure was obtained from internal sensors of the ventilator. An additional airway pressure signal and the three pleural pressure signals were measured using analogue pressure transducers. Pleural pressure signals were synchronized during post-processing with airway pressure, flow and volume using the redundantly measured airway pressure signal. Regional P_L_ in the three regions were calculated by subtracting the respective pleural pressure from airway pressure. Global P_L_ was calculated as mean P_L_ of the three sensors (ventral, dorsal and caudal). End-expiratory P_L_<2.9 cmH_2_O was defined as lung instability, as suggested elsewhere (Hedenstierna et al., 1981). In anaesthetized subjects in supine position, end-expiratory P_L_ of the left lung at closing capacity was 2.9 cmH_2_O.

Respiratory system elastance and resistance were determined using multiple linear regression of the linear equation of motion to airway flow, volume and pressure signals. Respiratory system MP was calculated by deriving mechanical energy (ME) per breath by numerical integration of the tidal pressure-volume-curve (PV curve) using a trapezoidal rule (Huhle et al., 2018).

### 2.3 Statistical analysis

All animals of the primary study were included in this analysis. Data are presented as mean and standard deviation (SD) if not stated otherwise. The statistical analysis was conducted with SPSS (Version 27, IBM Corp, United States). Significance was accepted at *p* < 0.05. Differences between TLVsupine and OLVsupine were tested using a t-test for paired samples. Differences between the respective body position during OLV and sequences of interventions were compared using a repeated measures linear mixed-effects model with OLVsupine, OLVsemilateral, OLVlateral, and OLVprone as within-subjects factor and with latin-square sequence as between-subjects factor. Pairwise *post hoc* multiple comparisons were performed when appropriate and corrected for multiple comparisons according to Šidák. Differences of the binary outcome lung instability, defined as end-expiratory P_L_ < 2.9 cmH_2_O (Hedenstierna et al., 1981) were tested using the χ^2^ test and pair-wise binomial *post hoc* test corrected according to Bonferroni.

## 3 Results

### 3.1 Global P_L_

Global transpulmonary pressures are depicted in Table 1. Global end-expiratory, end-inspiratory and ΔP_L_ were lower during TLVsupine than during OLVsupine. During OLV, end-expiratory P_L_ was lower in lateral as compared to supine and prone position, while end-inspiratory and ΔP_L_ did not differ among body positions.

**TABLE 1:** Global transpulmonary pressures.

Variable	BL	TLV supine	OLV supine	OLV semi-lateral	OLV lateral	OLV prone	TLVsupine vs. OLVsupine P =	Sequence P =	Position P =
P_L_ ee [cmH_2_O]	3.0 ± 1.6	2.4 ± 1.9	3.9 ± 1.9	2.8 ± 2.6	2.4 ± 2.0^S, P^	3.0 ± 1.7	0.004	0.113	0.007
P_L_ ei [cmH_2_O]	11.8 ± 2.3	13.4 ± 4	25.6 ± 5.9	24.3 ± 4.1	23.8 ± 4.5	23.5 ± 4.3	≤0.001	0.388	0.332
ΔP_L_ [cmH_2_O]	13.8 ± 2.5	15.9 ± 3.4	28.9 ± 5.3	28.6 ± 3.6	28.1 ± 4.0	29.5 ± 4.1	≤0.001	0.319	0.637

Mean ± SD; P_L_, transpulmonary pressure; ee, end-expiratory; ei, end-inspiratory, BL, baseline. S, *p* < 0.05 vs. OLVsupine; SE, *p* < 0.05 vs. OLVsemilateral; L, *p* < 0.05 vs. OLVlateral; P, *p* < 0.05 vs. OLVprone.

### 3.2 Regional end-expiratory P_L_

Regional end-expiratory P_L_ is depicted in Table 2. During TLVsupine, ventral and dorsal end-expiratory P_L_ were lower than during OLVsupine, while caudal end-expiratory P_L_ was not different.

**TABLE 2:** Regional transpulmonary pressures.

Variable	BL	TLV supine	OLV supine	OLV semi-lateral	OLV lateral	OLV prone	TLVsupine vs. OLVsupine P =	Sequence P =	Position P =
P_L_ ventral ee	3.5 ± 3.1	3.2 ± 2.7	4.8 ± 2.9 ^SE, L, P^	3.3 ± 2.9 ^L; P^	1.9 ± 3.3	1.7 ± 2.5	0.009	0.076	≤0.001
P_L_ dorsal ee	3.2 ± 1.5	2.4 ± 2.1	3.9 ± 2.1	3.7 ± 3.9	2.7 ± 2	4.3 ± 1.5	0.008	0.664	0.096
P_L_ caudal ee	2.4 ± 1.5	1.6 ± 2.1	3.1 ± 2.6	1.4 ± 2.3^L; P^	2.7 ± 1.7	3.3 ± 1.6	0.079	0.162	0.007
P_L_ ventral ei	12.2 ± 2.9	13.5 ± 3.7	25.4 ± 7	24.2 ± 3.9	23.6 ± 4.8	22 ± 4.7	≤0.001	0.316	0.089
P_L_ dorsal ei	11.6 ± 2.8	13.4 ± 4.1	25.7 ± 6	25.3 ± 5.2	24.2 ± 5.2	24.6 ± 4.3	≤0.001	0.612	0.708
P_L_ caudal ei	11.5 ± 2.7	13.2 ± 4.7	25.6 ± 5.8	23.5 ± 4.4	23.5 ± 4.5	23.9 ± 4.5	≤0.001	0.199	0.289

All values as Mean ± SD, in cmH_2_O; P_L_, transpulmonary pressure; ee, end-expiratory; ei, end-inspiratory, BL, baseline. S, *p* < 0.05 vs. OLVsupine; SE, *p* < 0.05 vs. OLVsemilateral; L, *p* < 0.05 vs. OLVlateral; P, *p* < 0.05 vs. OLVprone.

During OLV, dorsal end-expiratory P_L_ was not different among the body positions during OLV. Ventral end-expiratory P_L_ was higher in OLVsupine as compared to all other positions and higher in OLVsemilateral as compared with OLVlateral and OLVprone. Caudal end-expiratory P_L_ was lower in the semilateral position as compared to OLVlateral and OLVprone. Lung instability was detected more often in semilateral (26 out of 48 measurements; *p* = 0.012) and lateral (29 out of 48 measurements, *p* < 0.001) as compared to supine position (15 out of 48 measurements) and more often in lateral as compared to prone position (19 out of 48 measurements, *p* = 0.027).

### 3.3 Regional end-inspiratory P_L_ and regional driving P_L_

**FIGURE 2:**
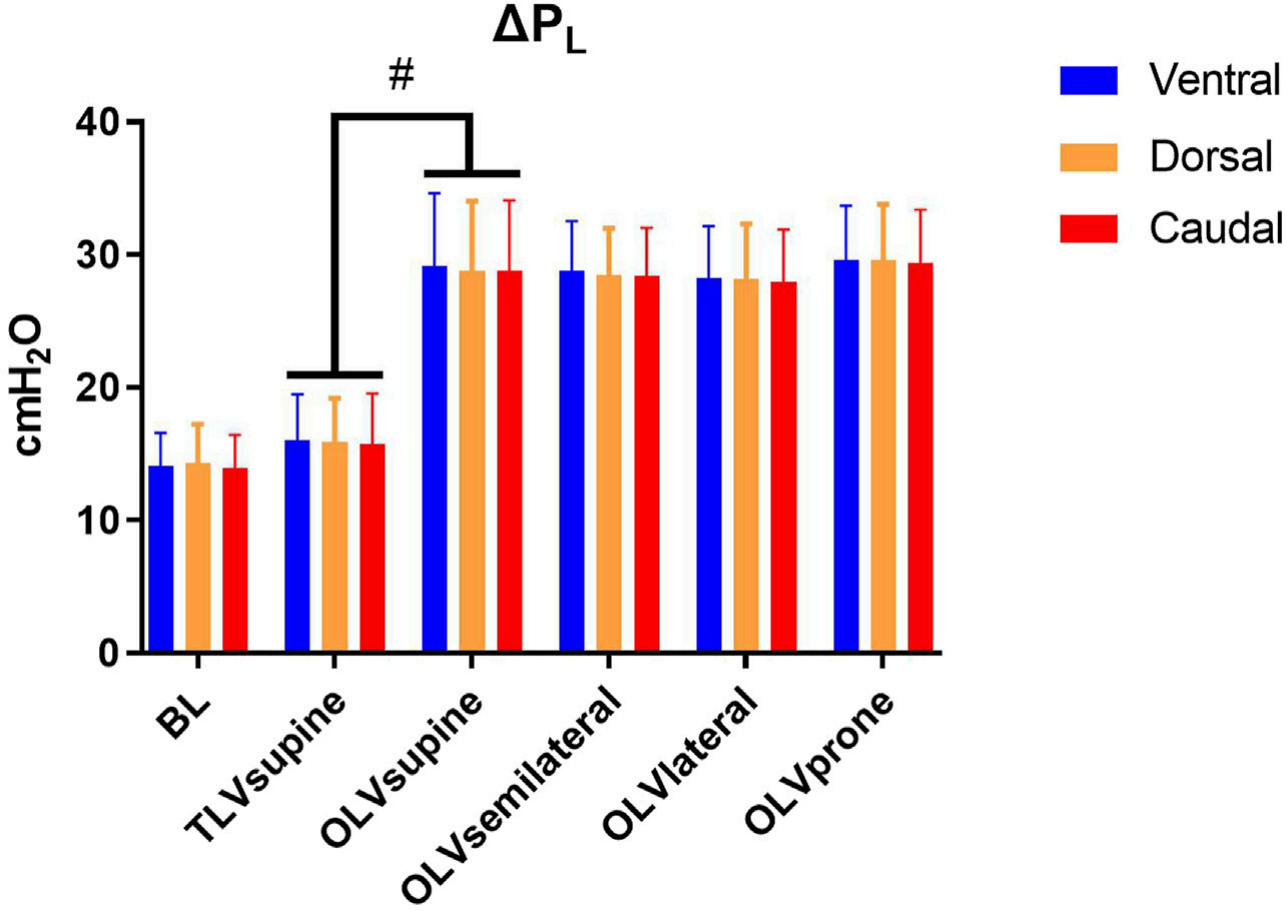
Regional transpulmonary driving pressure. Mean ± SD; ΔP_L_, transpulmonary driving pressure. Significance was accepted at *p* < 0.05. BL: Baseline. Differences between TLVsupine and OLVsupine were tested using a t-test for paired samples. Differences between the respective body position during OLV and sequences of interventions were compared using a linear mixed-effects model with repeated measures with OLVsupine, OLVsemilateral, OLVlateral, and OLVprone as within-subjects factor and with the sequence as between-subjects factor. Pairwise *post hoc* multiple comparisons were performed when appropriate and corrected for multiple comparisons according to Šidák (#*p* < 0.05).

Regional end-inspiratory P_L_ is depicted in Table 2. Ventral, dorsal, and caudal end-inspiratory P_L_ were lower during TLVsupine as compared with OLVsupine. During OLV, ventral, dorsal and caudal end-inspiratory P_L_ were not different between the different positions. During TLVsupine, ΔP_L_ was lower in all regions as compared with OLVsupine (Figure 2). During OLV, ΔP_L_ was not different among the body positions.

### 3.4 Respiratory system mechanics, mechanical work, and power

Respiratory variables are represented in Table 3. Respiratory rate, plateau, and mean airway pressure, as well as elastance and resistance, were lower during TLVsupine compared with OLVsupine, while there were no differences between positions during OLV. Respiratory system MW and MP were lower during TLVsupine as compared with OLVsupine. During OLV, MW and MP were not significantly different between the different body positions.

**TABLE 3:** Respiratory mechanics, mechanical work and power.

Variable	BL	TLV supine	OLV supine	OLV semi-lateral	OLV lateral	OLV prone	TLVsupine vs. OLVsupine P =	Sequence P =	Position P =
V_T_ [ml kg^-1^]	6.5 ± 0.3	6.4 ± 0.1	5.1 ± 0.2	5.1 ± 0.2	5.1 ± 0.2	5.1 ± 0.2	≤0.001	0.495	0.855
RR [min^-1^]	30 ± 2	29 ± 2	35 ± 0	35 ± 1	35 ± 0	35 ± 0	≤0.001	0.491	0.374
Pplat [cmH_2_O]	16.3 ± 2.7	18.5 ± 3.3	29.6 ± 5.9	28.9 ± 4.2	28 ± 4.8	29.3 ± 4.2	≤0.001	0.331	0.515
Pmean [cmH_2_O]	10.5 ± 0.9	11 ± 1.2	15.3 ± 1.7	15.3 ± 1.3	15 ± 1.3	15.7 ± 1.9	≤0.001	0.298	0.468
PEEP [cmH_2_O]	5.1 ± 0.1	5 ± 0.1	5 ± 0.2	5 ± 0.1	5 ± 0.2	4.9 ± 0.3	0.594	0.991	0.322
Auto-PEEP [cmH_2_O]	1.9 ± 0.6	1.3 ± 0.5	2.0 ± 0.9	1.9 ± 0.8	1.7 ± 0.9	2.2 ± 2.2	0.003	0.098	0.045
E [cmH_2_O L^-1^]	34.6 ± 8.5	42.6 ± 9.8	88.2 ± 23	84.8 ± 13.7	83.5 ± 16	86.3 ± 17	≤0.001	0.395	0.825
R [cmH_2_O s L^-1^]	11.5 ± 1.3	11.7 ± 1.7	24.6 ± 4.6	25.2 ± 3.8	24.3 ± 2.6	26.2 ± 5.6	≤0.001	0.473	0.355
MP [J min^-1^]	7.1 ± 1.8	7.8 ± 2	12.6 ± 2.2	12.9 ± 2.1	13 ± 2.2	13.2 ± 2.7	≤0.001	0.609	0.656
MW [J]	0.2 ± 0.1	0.3 ± 0.1	0.4 ± 0.1	0.4 ± 0.1	0.4 ± 0.1	0.4 ± 0.1	≤0.001	0.651	0.640

Mean ± SD; V_T_, tidal volume; Pplat, plateau airway pressure, Pmean, mean airway pressure; E, elastance of the respiratory system; R, resistance of the respiratory system; MP, mechanical power; MW, mechanical work; BL, baseline.

## 4 Discussion

The main findings of the present study were that, in anesthetized pigs, mean end-inspiratory and end-expiratory P_L_ and ΔP_L_ were higher during OLVsupine than TLVsupine as well as respiratory system elastance, resistance and MP. During OLV, only end-expiratory P_L_ was lower in semilateral and lateral compared to supine and prone position, mainly in ventral and caudal regions.

To our knowledge, this is the first comprehensive investigation on regional P_L_ during TLV and OLV in different body positions. Differently from esophageal manometry, which may overestimate P_Pl_ of non-dependent lung zones and underestimates P_Pl_ of dependent regions (Pelosi et al., 2001; Pelosi et al., 2011), we measured P_Pl_ locally. Thereby, the distribution of P_L_ in ventral, dorsal, and caudal region of the left hemithorax could be determined in presence of a contra-lateral open chest. Of note, mechanical ventilation settings both during TLV as well as OLV followed closely the clinical standard recommended by an expert panel-based consensus (Batchelor et al., 2019; Young et al., 2019).

### 4.1 Lung instability and stress

Our finding that end-expiratory P_L_ increased when switching from TLV to OLV in supine position might be explained by the fact that the pressure difference over the ribcage is reduced during pneumothorax as well as a shift of the mediastinal content towards the side of pneumothorax, reducing intrathoracic pressure. During OLV, ventral and caudal end-expiratory P_L_ differed depending on body position, with the lowest values in lateral and semilateral position, suggesting increased lung instability in this position. This finding is line with previous investigations in healthy volunteers during two lung ventilation (Ferris et al., 1959; Milic-Emili et al., 1964; Washko et al., 2006) and in thoracic surgery patients (Chiumello et al., 2020) using esophageal balloon manometry. Our finding can be explained by the fact that in the lateral position, the ventilated lung is compressed by the weight of the mediastinum, which shifts to the side of ventilation following gravity once the thorax is opened. More pronounced lung compression may increase the risk of end-expiratory lung collapse. As a consequence, potentially higher PEEP values are necessary to keep the lung open in lateral position. However, it is a matter of ongoing debate on which PEEP should be chosen during OLV (Battaglini et al., 2020). In addition to the set PEEP, auto-PEEP can influence end-expiratory P_L_. In our study however, auto-PEEP did not differ among the investigated body positions.

End-inspiratory P_L_, as well ΔP_L_, markedly increased in all regions of the lung during OLV as compared to TLV, theoretically indicating increased lung stress and tidal overdistension. High alveolar stress and overdistension are the driving forces of VILI (Güldner et al., 2016; Bates and Smith, 2018)*.* We used a low V_T_ during OLV, which is considered to be protective. However, even this low V_T_ resulted in high end-inspiratory P_L_ and high ΔP_L_, independently of the body position, putting the ventilated lung at risk of VILI during OLV. Our results might explain why the risk of postoperative pulmonary complications is increased after thoracic anesthesia (Uhlig et al., 2020).

### 4.2 Mechanical power and respiratory mechanics

The fact that, during OLV, MP was almost twice as high as during TLV is in line with the literature and likely explained by increased elastance. In a prospective, observational, single-center study in 30 patients, MP increased in the lateral position with OLV as compared to TLV (Chiumello et al., 2020). Interestingly, MP exceeded 12 J/min during OLV in our animals, a threshold that was associated with formation of lung edema during TLV in pigs (Cressoni et al., 2016). Furthermore, OLV was accompanied by an increase of elastance in our study, which means that the stress exerted on the parenchymal tissue increased, potentially leading to increased distension of the septal walls and VILI (Bates and Smith, 2018). The fact that elastance did not differ significantly among body positions contrasts with results reported during TLV elsewhere (Tanskanen et al., 1997). A likely explanation for this discrepancy is that, in our animals, elastance almost doubled during OLV compared to TLV, possibly masking less pronounced effects of body position. Furthermore, V_T_ adjusted to body weight during left lung ventilation was almost twice when adjusted for the relative size of the ventilated lung (assuming that the left lung accounts for approximately 45% of the total lung size), which may explain the increased elastance, despite the pneumothorax.

### 4.3 Potential implications of the findings

The findings of this study suggest that the added risk of VILI during of OLV, as compared to TLV, is explained by increased MP and lung distending pressures. Importantly, this was not amenable by protective low tidal volumes that are commonly used in clinical practice. It is worth noting that lateral and semi-lateral decubitus positon, the most common positions used during thoracic surgery with OLV, led to the highest risk of VILI, since they were accompanied by increased lung instability.

### 4.4 Limitations

The present study knows several limitations. First, the study was conducted as an exploratory sub-study of another trial and lacked formal sample size estimation. Second, we did not investigate the effects of right lateral and semilateral decubitus positions. A previous study reported lower end-expiratory P_Pl_, as estimated by oesophageal manometry, in the right lateral position compared to the left lateral position during OLV, which was explained by the lower volume of the left lung, thus leading to a smaller decrease in P_Pl_ during right decubitus compared to left decubitus position due to less traction of the smaller left lung on the pleural surface (Chiumello et al., 2020). Third, the cross-over design precluded assessment of lung injury. Therefore, we do not know whether lower end-inspiratory P_L_ during OLV in the lateral position increases the risk of VILI. Fourth, the thoracotomy limited the direct comparison of respiratory mechanics between TLV and OLV since the latter did not include assessment of chest wall mechanics. Fourth, the different shape of the rib cage and anatomical distribution of intrathoracic organs in pigs may lead to different amplitudes and distribution of pressures in humans.

### 4.5 Conclusions

In anesthetized pigs, protective low tidal volume OLV, as compared to TLV, increased lung stress and respiratory system MP and impaired respiratory system mechanics. Body position did not affect lung stress, or respiratory system mechanics and MP during OLV, but end-expiratory P_L_ was lowest in semilateral and lateral decubitus position.

## Data Availability

The raw data supporting the conclusion of this article will be made available by the authors, without undue reservation.

## Abbreviations

ANOVA, analysis of variance; FIO2, inspired fraction of oxygen; I:E, inspiratory to expiratory time ratio; LPS, Lipopolysaccharide; MP, mechanical power; MW, mechanical work; OLV, one-lung ventilation; PEEP, positive end-expiratory pressure; PL, transpulmonary pressure; PPl, pleural pressure; ΔPL, driving transpulmonary pressure; RR, respiratory rate; SD, standard deviation; TLV, two-lung ventilation; VILI, ventilator induced lung injury; VT, tidal volume.
